# TNF-alpha and IL-6 inhibit apolipoprotein A-IV production induced by linoleic acid in human intestinal Caco2 cells

**DOI:** 10.1186/s12950-015-0069-0

**Published:** 2015-03-22

**Authors:** Xiaoming Li, Min Xu, Min Liu, Yong Ji, Zongfang Li

**Affiliations:** National Local Joint Engineering Research Center of Biodiagnostics and Biotherapy, The Second Affiliated Hospital of Medical College, Xi’an Jiaotong University, Xi’an, 710004 China; Department of Pathology and Laboratory Medicine, University of Cincinnati, Cincinnati, 45237-0507 USA

**Keywords:** Apolipoprotein A-IV, TNF-α, IL-6, Fatty acid, Linoleic acid

## Abstract

**Background:**

Apolipoprotein A-IV (apoA-IV) is a protein mainly synthesized by enterocytes in the intestine. Its gene expression is suppressed during fasting and stimulated during active fat absorption. Chronic feeding of a high-fat (HF) diet abolishes the differential expression between fasting and fat-feeding and therefore may contribute to diet-induced obesity since apoA-IV is a potent satiety factor. It is well established that the circulating pro-inflammatory cytokines TNF-α and IL-6 are increased by HF feeding.

**Methods:**

To determine whether pro-inflammatory cytokines are involved in the diminished response of apoA-IV gene expression to fat-feeding, different concentrations of linoleic acid (LA), an important dietary fatty acid, was used to stimulate apoA-IV expression in human intestinal Caco2 cells. Cells were pre-treated with or without human recombinant TNF-α, IL-6 or their combination before the addition of LA. Real-time PCR and ELISA were used to detect and quantify RNA transcripts and proteins of apoA-IV and the cytokines.

**Results:**

LA stimulated gene and protein expression of apoA-IV in a dose and time dependent manner. Pre-treatment with the cytokines for 72 h significantly inhibited the increased expression of apoA-IV gene and protein induced by LA. Furthermore, the cytokines, especially TNF-α, also positively up-regulate the cytokine themselves in Caco2 cells.

**Conclusions:**

Our data indicate that the pro-inflammatory cytokines may be responsible for the reduced apoA-IV production in response to fat feeding. Because of apoA-IV’s role in satiety, we propose the inhibitory effect of circulating pro-inflammatory cytokines on apoA-IV production contributes to diet-induced obesity.

## Background

Obesity has reached epidemic proportions globally, and more than one-third of adults (35.7%) in USA are obese. Being overweight or obese increases the risk of diseases including cancer, coronary heart disease, type II diabetes, hypertension and stroke. Compelling evidence has demonstrated that dietary fat intake is a major cause of obesity, diabetes, and the associated metabolic syndromes [1,2].

Human apoA-IV is a 46-kDa protein produced by enterocytes of the small intestine and its production is stimulated by fat absorption [3]. ApoA-IV is secreted in association with triacylglycerol (TG) rich chylomicron particles. After entering the circulation, apoA-IV rapidly dissociates from chylomicrons and then transfers into high-density lipoproteins or becomes a lipoprotein-free fraction of the plasma in the postprandial state [4]. ApoA-IV has a number of well-established functions, including the inhibition of food intake, anti-inflammatory role, reverse cholesterol transport, and the regulation of glucose metabolism through the stimulation of insulin secretion and inhibition of hepatic gluconeogenesis [5-9]. Although the production of apoA-IV is stimulated by fat absorption, chronic ingestion of high fat (HF) feeding caused the adaptation of the intestinal apoA-IV response to lipid feeding. Weinberg et al. [10] first reported the adaptation of plasma apoA-IV in response to chronic feeding of a HF diet in humans. They observed that consumption of an HF diet for 1 week significantly elevated plasma apoA-IV, but after two weeks on the HF diet, the apoA-IV level returned back to baseline. Kalogeris and Painter [11] found that plasma apoA-IV increased initially by 40% in response to intra-gastric administration of fat emulsion within the first one to two days, but this was followed by the diminished responses with no increase in plasma apoA-IV levels by four days of fat feeding. Similarly, the jejunal mRNA levels and mucosal apoA-IV protein synthesis also showed time-dependent refractoriness to fat administration, suggesting both posttranslational (protein clearance) and/or pre-translation (transcriptional) adaptation of the intestinal apoA-IV production.

It is well established that circulating pro-inflammatory cytokines [e.g. tumor necrosis factor alpha (TNF-α), interleukin 6 (IL-6)] are induced in obesity and these pro-inflammatory cytokines play a crucial role in the development of metabolic syndrome [12-14]. Ding et al. reported that HF diet feeding and bacteria interaction promoted TNF-α mRNA production and intestinal inflammation in mice; and the increase TNF-α preceded obesity was strongly and significantly associated with progression of obesity and development of insulin resistance [15]. Recently, Ji et al. showed that intra-duodenal infusion of fat emulsion caused mucosal mast cells activation and increased lymphatic secretion of pro-inflammatory cytokines IL-6 [16]. These observations suggest that pro-inflammatory cytokines derived from gut may contribute to the development of obesity and associated metabolic syndrome.

It was reported that treatment with cytokines TNF-α or IL-6 in cultured pig hepatocytes decreased apoA-IV mRNA levels [17], suggesting that the cytokines may contribute to dietary-induced obesity through down-regulation of the important satiety signal apoA-IV expression [18]. To determine whether pro-inflammatory cytokines are involved in the attenuated response of apoA-IV to chronic HF feeding, we performed *in vitro* study to investigate the effect of human recombinant cytokines TNF-α, IL-6 or their combination on apoA-IV expression in response to the treatment of Linoleic acid (LA, an important dietary fatty acid) in cultured human intestinal Caco2 cells. Our results suggest that pro-inflammatory cytokines inhibit apoA-IV production induced by LA in Caco2 cells.

## Methods

### Materials

Linoleic acid, sodium taurocholate (TC) and other chemicals were obtained from Sigma-Aldrich (St. Louis, MO, USA). Recombinant human IL-6 (r-h-IL-6) and recombinant human TNF-α (r-h-TNF-α) were purchased from R&D Systems, Inc. (Minneapolis, MN, USA). Dulbecco’s modified essential medium (DMEM), fetal bovine serum (FBS) and antibiotic: antimycotic mixture were obtained from Thermo Fisher Scientific (Carlsbad, CA, USA).

### Cell cultures

Caco-2 cells, obtained from the American Type Culture Collection (ATCC, Rockville, MD, USA), were grown in DMEM containing high glucose, 20% FBS, and a 1% antibiotic: antimycotic mixture. 70-80% confluent Caco-2 cells were plated onto 24-well plastic dishes (Becton-Dickinson Labware, Lincoln Park, NJ, USA) at initial densities of 1 × 10^5^ cells/well in complete growth medium. The medium was changed every other day for 14–21 days. This procedure is known to induce differentiation of Caco-2 cells into more enterocyte-like cells [19].

### LA and cytokine treatment to Caco2 cells

To prepare LA stocks (20 ×), 20 mM LA and 10 mM TC were mixed and stored at −20°C. To prepare TNF-α and IL-6 (1000 ×, 10 mg/ml) stocks, r-h-IL-6 and r-h-TNF-α were dissolved in phosphate buffered saline (PBS). The differentiated Caco2 cells were incubated in growth medium with or without 20 ng/ml TNF-α, or 20 ng/ml IL-6, respectively, or a mixture of the both (20 ng/ml/each) for the indicated time, and then changed into DMEM/ high glucose/1% FBS with or without indicated amount of LA: TC for the time indicated, with 0.5 mM TC as vehicle control. The cells were harvested and lysed, and the total RNA was isolated.

### Real-time RT-PCR

Total RNA was isolated from cells with RNeasy Mini Kit (Qiagen, Germantown, MD, USA). First-strand cDNA was synthesized from 1 μg total RNA with Scripts™ cDNA Synthesis Kit (Bio-Rad Laboratories Inc., Hercules, CA, USA) according to the manufacturer’s instruction. Real-time PCR was performed using iQ SYBR Green Supermix (Bio-Rad Laboratories Inc. Hercules, CA, USA) with an iCycler iQ Detection System (icycler iQ. Multicolor Real-time PCR Detection System, Bio-Rad) and normalized to β-actin. All primers were purchased from Integrated DNA Technologies (Coralville, IA,USA). PCR primer pairs used were: human *apoA-IV* forward, 5′-ACCCAGCTAAGCAACAATGC-3′, and reverse, 5′-TGTCCTGGAAGAGGGTACTGA-3′; human *Il6* forward, 5′-TGATGGATGCTACCAAACTGG-3′, and reverse, 5′-TTCATGTACTCCAGGTAGCTATGG-3′; human *Tnf*α forward, 5′-TCTTCTCATT CCTGCTTGTGG-3′, and reverse, 5′- GGTCTGGGCCATAGAACTGA-3′; human *apoC-III* forward, 5′-AGACCGCCAAGGATGCACTGA-3′, and reverse, 5′-TCTGACCTCAGGGTCCAAATCC-3′; *βActin* forward, 5′-TTGCTGACAGGAT-GCAGAAGGAGA-3′, and reverse, 5′-TCAGTAACAGTCCGCCTAGAAGCA-3′.

### Enzyme linked immunosorbent assay (ELISA)

ELISA was performed using Human ApoA-IV ELISA Kit from Millipore Corporation (Billerica, MA, USA), Human IL-6 and Human TNF-α ELISA Kits from RayBiotech (Norcross, GA, USA) according to the protocol provided by the manufacturer. The culture mediums collected from the cells treated with LA with or without cytokines were used to measure the proteins released from the cells by ELISA.

### Statistics

Data represent Mean ± SE from three or four wells in each experiment of at least two independent cell culture experiments. Significance of differences was determined by one-way ANOVA or two-way ANOVA followed by Tukey test method. P value less than 0.05 was considered significant.

## Results

### LA stimulates *apoA-IV* gene expression in Caco2 cells

**Figure 1:**
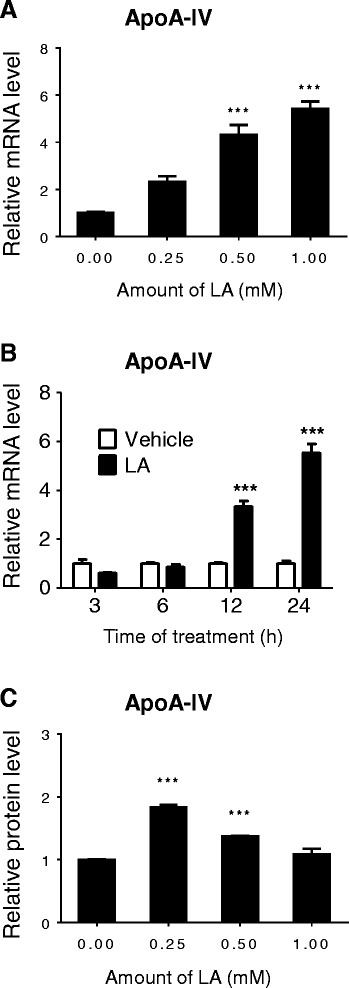
**LA induced** ***apoA-IV*** **gene expression in Caco2 cells.** Differentiated Caco2 cells were supplemented with or without different amount of LA for 24 h **(A)** or for the indicated time with 1 mM LA **(B)** up to 24 h. The *apoA-IV* mRNA levels were measured by real time RT-PCR. The levels of apoA-IV protein released into culture medium from the cells were measured by ELISA **(C)**. ***P <0.001 *vs.* TC vehicle control.

To induce gene expression of *apoA-IV* in Caco2 cells, the medium of differentiated Caco2 cells was supplemented with LA mixed with TC in the indicated dose and time, as described previously [19]. First, we studied the effect of different concentrations (0.25, 0.5, 1.0 mM) of LA on *apoA-IV* gene expression after 24 h incubation. As shown in Figure 1A, 0.25 mM of LA was able to enhance *apoA-IV* gene expression by 1.31 fold, compared with TC vehicle control, although there was no statistically significant difference. 0.5 mM of LA significantly increased *apoA-IV* mRNA level by 3.3 fold, and 1 mM of LA by 4.42 fold. Second, we determined the time course of *apoA-IV* gene expression of Caco2 cells after incubation with 1 mM LA for various times (3, 6, 12, and 24 h). As shown in Figure 1B, *apoA-IV* mRNA level started to increase by 2.34 fold when incubated with LA for 12 h, and increased by 4.42 fold at 24 h, compared with that treated with vehicle controls. Finally, to test whether the apoA-IV protein is also changed with its mRNA levels, we measured the apoA-IV protein levels in the culture medium after the treatment with different amount of LA for 24 h incubation. As presented in Figure 1C, 0.25 mM LA surprisedly showed a maximum effect on apoA-IV protein production, which was stronger than 0.5 mM LA. However, when LA dose increased to 1 mM, its effect on apoA-IV was almost gone. These data indicate that LA up-regulates both apoA-IV mRNA and protein levels in dose-dependent manner, but in different pattern, in the Caco2 cells.

### Cytokines inhibit apoA-IV production induced by LA

**Figure 2:**
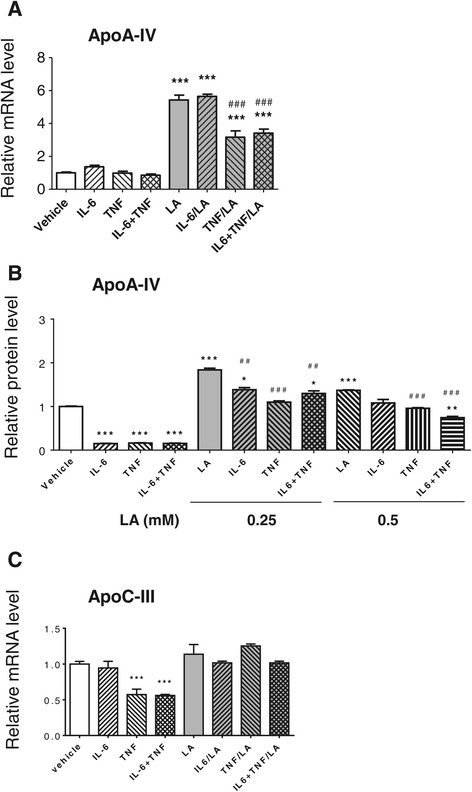
**Effects of cytokines on** ***apoA-IV*** **and** ***apoC-III*** **gene expression. (A)** Effects of LA and cytokines on *apoA-IV* gene expression. Differentiated Caco2 cells were pre-treated with or without r-h-TNF-α (20 ng/ml) or r-h-IL-6 (20 ng/ml) or the combination of these two cytokines for 72 h before changing into medium with or without 1 mM LA for 24 h. **(B)** Effects of LA and cytokines on apoA-IV protein secretion. Differentiated Caco2 cells were pre-treated with or without r-h-TNF-α (20 ng/ml) or r-h-IL-6 (20 ng/ml) or the combination of these two cytokines for 72 h before changing into medium with or without 0.25 or 0.5 mM LA for 24 h. The apoA-IV proteins in culture medium secreted from the cells were measured by ELISA. **(C)** Effects of LA and cytokines on *apoC-III* expression. The samples were processed at the same ways described at (A). The mRNA levels of both *apoA-IV* and *apoC-III* were measured by real time RT-PCR. * *P* < 0.05, ** *P* < 0.01, *** *P* < 0.001 *vs*. vehicle control; and ^##^ *P* < 0.01 ^###^ *P* < 0.001 *vs.* LA.

It has been reported that cytokine IL-6 and TNF-α reduced *apoA-IV* mRNA levels in pig hepatocytes [17]. To determine whether long time exposure to TNF-α and IL-6 also affects *apoA-IV* gene expression induced by LA in Caco2 cells, the differentiated Caco2 cells were pre-treated with r-h-TNFα and r-h-IL6 for 72 h, followed by incubation with or without 1 mM LA for 24 h. As shown in Figure 2A, the cytokines did not affect *apoA-IV* mRNA in the cells without LA treatment. However, pre-treatment with the combination of these two cytokines significantly attenuated LA-induced increase of mRNA levels (Figure 2A) and apoA-IV protein secretion (Figure 2B). To further determine which cytokine, TNF-α or IL-6, exerts the inhibitory effect, we pre-incubated the cells with either TNF-α or IL-6, respectively, followed by the LA treatment. We found that TNF-α significantly attenuated the increased levels of both *apoA-IV* mRNA (Figure 2A) and protein (Figure 2B) induced by LA, whereas IL-6 had no such effect on apoA-IV mRNA level (Figure 2A), but significantly attenuated apoA-IV protein level induced by 0.25 mM LA (Figure 2B). Furthermore, TNF-α and IL-6 themselves decreased apoA-IV protein levels (Figure 2B), but not *apoA-IV* mRNA levels (Figure 2A). These data indicate that both TNF-α and IL-6 inhibit the stimulatory effect of LA on apoA-IV production.

### Effects of LA and cytokines on *apoC-III* gene expression in Caco2 cells

*ApoA-IV* is a member of the *apoA-I/C-III/A-IV* gene cluster. It was reported previously that intestinal expression of *apoA-IV* and *apoC-III* is coordinately regulated by dietary lipid in newborn swine [20]. To test whether LA specifically stimulates *apoA-IV* expression in Caco-2 cells, we measured the mRNA level of *apoC-III* in the presence of LA or/and the cytokines, and determined whether the pre-treatment of cytokines affects the responses of *apoC-III* to LA. As shown in Figure 2C, treatment with 1 mM LA alone or pre-treatments with cytokines (IL-6, TNF-α, or their combination) did not significantly affect *apoC-III* gene expression in the Caco-2 cells. Interestingly, TNF-α, but not IL-6, significantly decreased *apoC-III* expression in the Caco-2 cells (Figure 2C). These observations indicate that LA specifically stimulates *apoA-IV* expression in Caco-2 cells. In addition, different from the changes in *apoA-IV* mRNA levels, *apoC-III* mRNA was significantly decreased by TNF-α at the condition without LA stimulation.

### LA stimulates productions of *Il6* and *Tnfα*

**Figure 3:**
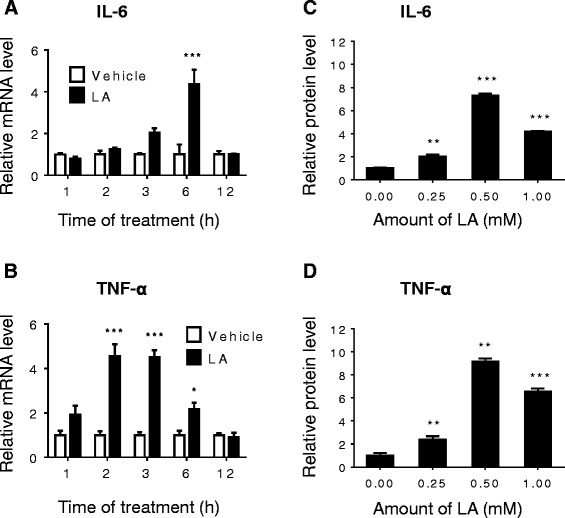
**LA stimulated gene expression of** ***Il6*** **and** ***Tnfα*** **in Caco2 cells.** Differentiated Caco2 cells were supplemented with or without 1 mM LA for the indicated times for mRNA measurement or with different amount of LA for 24 h for protein measurement. The mRNA levels of *Il6* and *Tnfα* were measured by real time RT-PCR **(A and B)**. Their proteins from culture medium were measured by ELISA **(C and D)**. *P < 0.05, ** *P* <0.01, *** *P* <0.001 *vs.* vehicle control.

**Figure 4:**
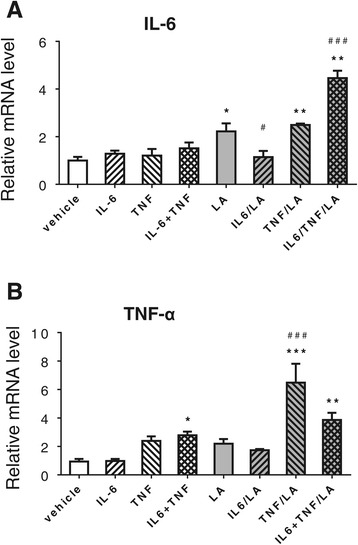
**Effect of cytokines on gene expression of** ***Il6*** **(A) and** ***Tnfα*** **(B) in Caco2 cells.** Differentiated Caco2 cells were pre-treated with or without r-h-TNF-α (20 ng/ml), r-h-IL-6 (20 ng/ml) or their combination for 72 h before changing into the medium with or without LA (1 mM) for 24 h. The mRNA levels of both *Il6* and *Tnfα* were measured by real time RT-PCR. *P < 0.05, **P < 0.01, ***P < 0.001 *vs.* vehicle control; and ^#^ *P* <0.05, ^###^ *P* <0.001 vs*.* LA.

It has been reported that long-chain fatty acid, e.g. the LA, can enhance *Il6* production in rat intestinal epithelial cells [21]. To determine whether LA is able to affect gene expressions of endogenous cytokines IL-6 and TNF-α in the Caco2 cells, we measured mRNA levels of *Il6* and *Tnfα* at the indicated times of LA treatment from Caco2 cells and their proteins released from the cells after the treatment with different amount of LA for 24 h. We found that the LA stimulated *Tnfα* and *Il6* gene expressions sooner than *apoA-IV* expression. The mRNA level of *Il6* started to increase by 1.03 fold after 3 h treatment, although the difference is not statistically significant, and then reached to 3.4 fold increase at 6 h (Figure 3A). The mRNA level of *Tnfα* was significantly increased sooner than *Il6* mRNA and it reached to 3.57 fold at 2 h, 3.52 fold increases at 3 h and then reduced to 1.4 fold increases by 6 h (Figure 3B). It is noteworthy that the pattern of *Tnfα* gene expression was similar to *Il6*, and all dropped back to basal level at 12 h, but *Il6* start to express again at 24 h post-treatment of LA (Figure 4A), which could be positive feedback regulation from the other factors, such as TNF-α, as a result from LA stimulation. We also measured their protein levels and found that 0.5 mM LA had the maximum effects on both IL-6 and TNF-α protein levels although 0.25 mM and 1 mM LA increased the cytokine protein productions, too (Figure 3C and D).

### Cytokines positively regulate their own gene expression

To determine whether the cytokines also affect their own expression, the mRNAs of *Il6* and *Tnfα* were measured in the Caco-2 cells treated with or without the cytokines and/or with LA. As shown in Figure 4, no change in *Il6* mRNA levels was found in the cells when these two cytokines were treated individually or their combination without LA treatment. However, LA significantly stimulated *Il6* gene expression, which was significantly attenuated by pre-treatment of IL-6, but not by TNF-α. Additionally, pre-treatment with the combination of IL-6 and TNF-α led to further increase of *Il6* gene expression in those cells. Similarly, treatment with TNF-α, especially the combination of IL-6 and TNF-α, stimulated *Tnfα* gene expression in the Caco-2 cells either with or without LA treatment. These data indicate that the cytokines, especially TNF-α, could positively up-regulate their own gene expression in Caco-2 cells.

## Discussion

ApoA-IV is produced dominantly in the gut and has many physiological functions which include satiety, regulation of glucose metabolism, reverse cholesterol transport and also anti-inflammation [6,8,9,22]. Therefore, the reduced apoA-IV production in response to active lipid absorption after chronic HF diet-feeding has been implicated in the diet-induced obesity and metabolic disorders. The purpose of this study was to test the hypothesis that the inflammatory cytokines expressed and released during chronic HF feeding potentially play an important role in this reduced apoA-IV response to fat absorption. Linoleic acid (LA) is an important dietary fatty acid and a major component in many HF diets or the lipids that have been used to induce apoA-IV production [11,23].

Caco-2 cells, a human colon carcinoma cell line, have been widely used as a model to study intestinal epithelial cell metabolism, including lipids and lipoproteins [19,24-27]. However, it has not been tested previously whether LA stimulates apoA-IV expression in those cells. With different dose of LA and varying incubation times with the differentiated Caco2 cells, for the first time, we found that *apoA-IV* gene expression and protein secretion was significantly increased in response to LA stimulation. Notably, apoA-IV production was significantly enhanced with lower amount of LA (0.25 mM) stimulation, but high dose of LA (1 mM) failed to increase apoA-IV protein production, although *apoA-IV* mRNA was significantly increased with this amount of LA from 12 h to 24 h. It was well-known that fat induces both apoA-IV synthesis and secretion from the gut, but up to now, its molecular mechanism is not clear. Our observations that apoA-IV mRNA level was increased with the increase of LA amount, but protein secretion of apoA-IV could not induced by a higher amount of LA, indicating that higher amount of LA may activate or induce other related factors, e.g. IL-6 and TNF-α, which can impact apoA-IV’s translation and the stability.

*ApoA-IV* is a member of a closely linked *apoA-I/C-III/A-IV* gene cluster, a target for acute phase proteins. A coordinately regulation of *apoC-III* and *apoA-IV* expression by lipids has been previously described [20]. However, it is also reported that the regulation of the members of this cluster by fat and inflammatory processes in the gut is not shared by all members of the cluster, e.g. neither the magnitude of response to the stimuli, nor the behavior of apoA-IV and apoC-III was similar [17]. Our data showed no difference in *apoC-III* gene expression in the cultured Caco2 cells treated with LA, IL-6 or the both together. In contrast, significant change in *apoA-IV* gene expression was observed when the Caco2 cells were exposed to the same cytokines. These results indicate a loss of the coordinated response reported previously in the animals [20], and suggest a complex interplay of transcription factors modulated by species-specific signaling pathways in response to a specific stimuli.

Our data also showed that LA not only induced *apoA-IV* gene expression, but also stimulated TNF-α and IL-6 secretion in Caco2 cells. Circulating cytokines, such as TNF-α or IL-6, have been shown to be elevated in obese humans [28,29] and this could be reversed with weight loss [30]. While the mechanisms underlying obesity-associated inflammation are not fully understood, a number of studies suggested that the inflammation may derive from the accumulation of activated macrophages within adipose tissue, liver, and the enlarged adipocytes in obese animals and humans [14,31,32]. Recent studies [15,16] suggest that 1) the gastrointestinal tract is another and early source of inflammation associated with diet, 2) the onset of intestinal inflammation precedes diet-induced increases in body weight, fat mass, as well as insulin resistance, and 3) the degree of TNF-α induction strongly correlates with diet-induced increases in weight, adiposity, plasma glucose, and insulin. Our data that LA-induced elevation of both *TNF-α* and *IL-6* mRNAs and of protein levels in Caco2 cells further support the concept that inflammatory cytokines released from the gut induced by fatty acid is the early source of inflammation associated with the development of obesity and/or metabolic disorders because TNF-α and IL-6 are widely used as early biomarkers of inflammation of insulin resistance and the obesity relative diseases.

It has been reported that TNF-α activates NF-κB and other inflammatory pathways [33,34]. As we described above, LA not only induced *apoA-IV* gene expression but also stimulated the gene expression and secretion of the cytokines. In addition, consistent with previous studies, TNF-α and IL-6, especially TNF-α, are further stimuli in the expression of the cytokines in the Caco2 cells, and enhance the effect of LA on these cytokines.

It rapidly became clear that early proinflammatory effects of HF diet, such as elevated TNF-α, could serve as a trigger for subsequent inflammation or insulin resistance through activating NF-kB and other inflammatory pathways involved in the etiology of insulin resistance and Type 2 diabetes. In addition to regulating metabolic homeostasis and intracellular signaling pathways that are well-established metabolic functions, inflammatory cytokines can impact on release of gut hormones such as glucagon-like peptide 2 (GLP-2) [15]. Our observations that these two cytokines reduced apoA-IV production, but not *apoA-IV* gene expression, imply that these cytokines’ inhibitory effects on apoA-IV not only occur at transcriptional levels but also at translational or posttranslational levels.

Pro-inflammatory molecules released by intestines, adipose and liver enter the circulation to influence the expression of apoA-IV at the gut. Our data that exogenous cytokines decreased apoA-IV production induced by LA support our hypothesis that pro-inflammatory cytokines are involved in reducing apoA-IV responses to fat feeding. These results support the notion that cytokines induction by chronic fat feeding is responsible for the blunted apoA-IV production normally stimulated by active fat absorption.

## Conclusions

Our study demonstrates that fatty acid LA induces *apoA-IV* gene expression and, at the same time, promotes inflammatory cytokines expression in the Caco2 cells. After chronic HF diet-feeding, constant and increased cytokine release resulted from positive feedback regulation of the cytokines suppresses apoA-IV production in response to fat absorption. Because of apoA-IV’s roles in the regulation of food intake, lipid absorption and glucose metabolism, the correlation between the local/circulating pro-inflammatory cytokines and apoA-IV production contributes to diet-induced obesity and the associated disorders.

## Abbreviations

LA: Linoleic acid
ApoA-IV: Apolipoprotein A-IV
TNF-α: Tumor necrosis factor alpha
IL-6: Interleukin-6
HFD: High fat diet
TG: Triacylglycerol
ApoC-III: Apolipoprotein C-III
